# Detection of *COPB2* as a *KRAS* synthetic lethal partner through integration of functional genomics screens

**DOI:** 10.18632/oncotarget.16079

**Published:** 2017-03-10

**Authors:** Eleni G. Christodoulou, Hai Yang, Franziska Lademann, Christian Pilarsky, Andreas Beyer, Michael Schroeder

**Affiliations:** ^1^ Biotechnology Center, TU Dresden, Dresden, Germany; ^2^ Department of Medical Oncology, National Cancer Center of Singapore, Singapore; ^3^ Chirurgische Klinik, Translational Research Center, Universitätsklinikum Erlangen, Erlangen, Germany; ^4^ Medizinische Fakultät Carl Gustav Carus, TU Dresden, Dresden, Germany; ^5^ Cellular Networks and Systems Biology, University of Cologne, Cologne, Germany

**Keywords:** KRAS, synthetic lethal partner, ranking, rank aggregation, functional screens

## Abstract

Mutated *KRAS* plays an important role in many cancers. Although targeting *KRAS* directly is difficult, indirect inactivation via synthetic lethal partners (SLPs) is promising. Yet to date, there are no SLPs from high-throughput RNAi screening, which are supported by multiple screens. Here, we address this problem by aggregating and ranking data over three independent high-throughput screens. We integrate rankings by minimizing the displacement and by considering established methods such as RIGER and RSA.

Our meta analysis reveals *COPB2* as a potential SLP of *KRAS* with good support from all three screens. *COPB2* is a coatomer subunit and its knock down has already been linked to disabled autophagy and reduced tumor growth. We confirm *COPB2* as SLP in knock down experiments on pancreas and colorectal cancer cell lines.

Overall, consistent integration of high throughput data can generate candidate synthetic lethal partners, which individual screens do not uncover. Concretely, we reveal and confirm that *COPB2* is a synthetic lethal partner of *KRAS* and hence a promising cancer target. Ligands inhibiting *COPB2* may, therefore, be promising new cancer drugs.

## INTRODUCTION

### *KRAS* and cancer

*KRAS* (Kirsten rat sarcoma viral oncogene homolog) is a protooncogene, whose constitutive activation through point mutations drives the neoplastic transformation in many cancers. Somatic *KRAS* mutations are frequent inleukemia, ovarian, colon, thyroid and lung cancers [1]. *KRAS* is one of the most frequently activated oncogenes, with 17 to 25% frequency among all human tumors. 90% of pancreatic tumors harbor an activating *KRAS* mutation. KRAS is a small GTPase molecule, which acts as a GTP to GDP converter in its wild type state. When KRAS is in its active or GTP-bound state, it contributes to the propagation of growth factor signals from the extracellular environment to the nucleus. Growth factors are responsible for stimulating processes important to the cell, such as growth, proliferation, healing, and differentiation. Normally, *KRAS* is inactivated again by GAP. However, mutated *KRAS* loses this capability and remains locked in its active state. Upon activation, *KRAS* drives the RAS-MAPK pathway leading to uncontrolled proliferation. *KRAS* is considered to be largely undruggable [2, 3] and despite recent successes [4] patients with *KRAS* mutations still have very poor prognosis.

### Synthetic lethality

Due to the difficulty of directly inhibiting *KRAS*, it has been proposed to repress its synthetic lethal partners [5]. Two genes are synthetic lethal if their simultaneous perturbation results in death, whereas a perturbation of just one of the two does not [6]. Concretely, a synthetic lethal partner (SLP) of *KRAS* is lethal to the cell if the cell has mutated *KRAS*, but it is not lethal in a cell with wild-type *KRAS*.

Therapeutic strategies leveraging synthetic lethality have recently been brought to clinical trials with encouraging first results [5]. The most promising ex-ample is the synthetic lethal interaction of *PARP* and *BRCA1/BRCA2* in cases of ovarian and breast cancer, which was very successful in phase II clinical trials [7]. Another study has recently demonstrated success in simultaneously targeting two genes interacting with *KRAS* [8], which further increases the complexity of the search space for new treatment options.

### RNAi screens for *KRAS* SLPs show little overlap

In the past, several knock-down approaches identified candidate *KRAS* SLPs (e.g. [9–14]; see Supplementary Information for a complete list). Conceptually, all of these screens are similar: several genes in cells with and without activating *KRAS* mutations are knocked-down and then screened for inhibitions that preferentially kill *KRAS* mutated cells. The major limitation of this approach is the inconsistency between the experimental results. Supplementary Table 1 lists some 70 *KRAS* SLPs, but there is no SLP confirmed by all of the screens and studies. A mere seven SLPs (proteasome components *APC/C, PSMA5, PSMB5, PSMB6 and PSMD14*, as well as *BIRC5*, and *GATA2*) are shared between two studies. Such inconsistencies arise due to the use of cell lines with different genetic backgrounds, use of different RNAi libraries, or due to different assays for quantifying the SLP phenotype. For example, different RNAi constructs targeting the same genes could differ with respect to their off-target effects [15, 16], leading to deviating phenotypes.

### Robust SLPs though consistent data integration across screens

**Figure 1:**
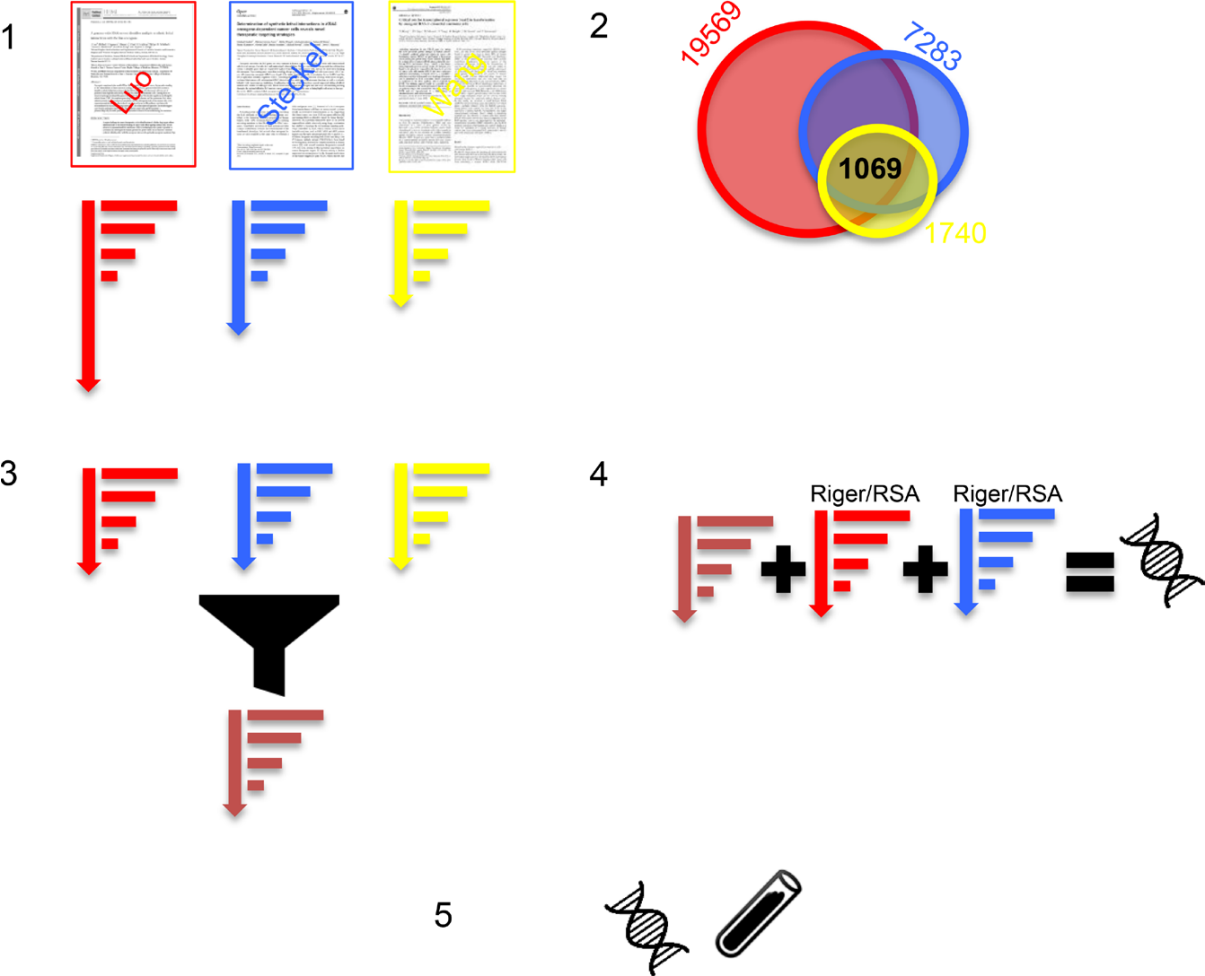
(**1**) We identified three *KRAS* SLP RNAi screens (Luo, Steckel, Wang) and normalized and ranked the genes per screen. (**2**) The three screens share 1069 genes. (**3**) The three rankings are aggregated by minimal displacement into one new optimized ranking (brown). (**4**) The ranking is combined with the established Riger and RSA ranking methods leading to a novel predicted SLP that is consistently high across screens and methods. (**5**) The predicted SLP is experimentally tested in a knock-down experiment.

Despite these inconsistencies, we hypothesize that a consistent aggregation and re-ranking can lead to SLP candidates, which are supported by multiple screens and are hence more robust. Therefore, we set out to combine results from multiple screens using a computational framework that specifically identifies genes consistently showing SLP effects across screens. The framework accounts for variable sizes of the screens (numbers of genes targeted), variable numbers of cell lines used, and different noise levels in the screens and is therefore robust. Consider Figure 1: First, we identify three relevant screens and normalize and rank screens individually. Second, we find 1069 genes common to all three screens. Third, we integrate the three rankings minimizing overall displacements of genes. Forth, we combine the aggregated ranking with other, established methods (Riger and RSA) leading to a robust prediction consistent across screens and consistent across methods. Finally, the prediction is experimentally validated.

## RESULTS

### Selected screens with minimal experimental variation share 1069 genes

Three large-scale RNAi screens were selected for prioritizing robust SLP candidate genes [10–12] (see Table 1 [10–12] and Figure 1).

**Table 1:** RNAi datasetes

Author	Genes	Isogenic	Cell lines	
Luo, Cell, 2009 [10]	19569	2 isogenic	DLD1 *KRAS*^+/−^ *KRAS*^+/G13D^ mutant	wild-type DLD1
Steckel, Cell Res., 2012 [11]	7283	2 isogenic	HKE3 *KRAS*^+/+^ *KRAS*^+/G13D^ mutant	wild-type HCT116
Wang, Oncogene, 2010 [12]	1740	2 isogenic	HKE3 *KRAS*^+/+^ *KRAS*^+/G13D^ mutant	wild-type HCT116

Three RNAi screens for *KRAS* synthetic lethal partners serve as base for the present meta analysis.

Subsequently, we refer to these screens based on their first author's names as Luo, Wang, and Steckel. These screens were chosen because they were performed on isogenic cell lines, which minimizes variance due to the genetic background, and because all cell lines originate from colorectal cancer with a G13D mutation, which further increases the similarity of the targeted cells. The Luo and Wang screens measured the impact of 1-15 (average 3) and 3 independent shRNAs per gene, respectively. Steckel, on the other hand, measured the impact of pools of 4 siRNAs per gene on caspase activity as a readout for apoptosis. Hence, whereas the Luo and Wang screens provide one cell viability score per shRNA (i.e. multiple per gene), the Steckel screens only reports one value per gene. Further, the Luo and Wang screens were performed in three replicates each, whereas only one replicate per gene is available from the Steckel screen (Table 1). All subsequent analyses were performed using the 1069 genes commonly targeted by all three screens. Due to the gene selection in the original screens, these 1069 genes are enriched for druggable kinases and for known cancer genes. Data from the Luo and Steckel screens had already been normalized by the authors using the mean and median absolute deviation (MAD), respectively. Data from the Wang screen were normalized by us using MAD [17]. Subsequently, individual gene scores were computed as follows.

In the case of the Steckel screen, where only summarized per sample and per gene measurements are available, we selected the genes that induce a *KRAS* - mutant cell specific apoptosis rate, which is higher than the median +1 *KRAS* -mutant apoptosis rate. In case of the other two screens, where replicates were available, we computed the average difference in cell viability between *KRAS* mutants and *KRAS* wild type cells (averaged across siRNA constructs) and determined the significance of that difference using the paired *t*-test. We refer to this initial data processing as Standard Method. More details are provided in the Methods section.

### Poor consistency between screens

As discussed above, the three screens have a similar experimental set- up and they all aim to find SLPs of the G13D *KRAS* mutation. Yet, when ranking the screens individually using the Standard method (see Methods), there is poor agreement. The three screens share only three genes (*BACH2, FOS, COPB2*) within the top 10% of their rankings. Among any two screens there are between 15 to 66 genes in common. In fact, only the intersection of the Luo and Wang screen is significant (*p*-value of 0.0123, Fisher's exact test). The intersections of Luo/Steckel and Wang/Steckel are not better than chance (*p*-value greater 0.08, Fisher's exact test). The slightly better *p*-value for Luo/Wang might result from replicate measurements, which likely improved the stability of the results. Nonetheless, the screens do not agree.

### Luo ranks known SLPs high, steckel and wang don’t

**Figure 2:**
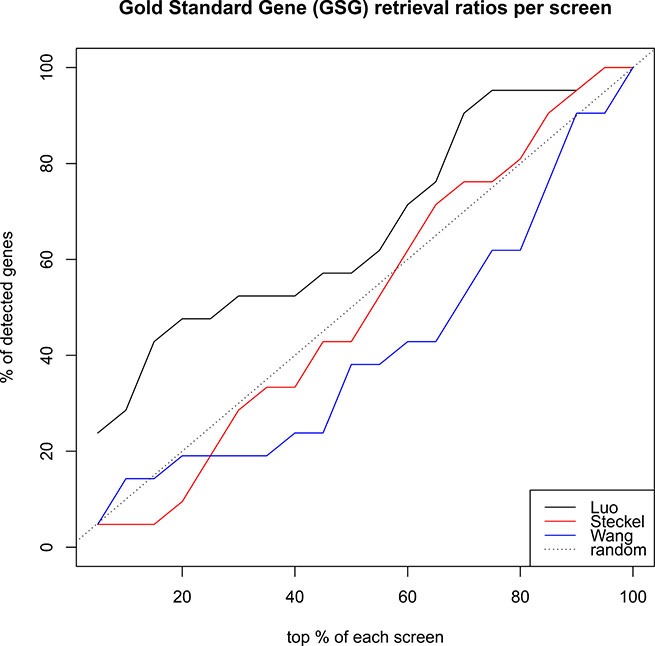
How well do rankings from screens recover the 21 known SLPs (gold standard), which were part of the screens? Luo (black) performs better than random, while Wang and Steckel perform slightly worst. Thus, more weight will be given to the Luo screen than to the Steckel and Wang screens.

In order to further evaluate the quality of the screens we assembled 28 known SLPs from literature (Supplementary Table 1). 21 of these SLPs were common to all three screens and are subsequently referred to as ’Gold Standard Genes’ (GSGs). We investigated the ranking of these GSG in each screen (Figure 2). In a successful screen, GSGs should rank high. However, Luo was the only screen enriching GSGs at the top of the list. These findings further underline the considerable variability inherent in the screens.

### Rank aggregation with spearman's footrule

Consider Figure 1. In the third step, the three selected screens are aggregated. In order to obtain such an aggregated gene ranking that is maximally consistent between the screens, we re-rank the genes such that the deviation of the aggregated ranking is minimal with respect to the three original rankings. Here, we use Spearman's footrule to quantify differences between rankings.

Spearman's footrule calculates the difference between a set of ranked lists by measuring the total number of displacements for an element (here gene) across the lists. The RankAggreg algorithm aims at minimizing Spearman's footrule between the input lists and the output aggregated list [18]. Please see Methods for more details on Spearman's footrule distance.

### Weighted rank aggregation on luo, wang, and steckel

We ranked the Luo, Wang, and Steckel screens using the standard method (see Methods) and applied the above rank aggregation procedure. To reflect the screens’ performance in recovering known SLP from the gold standard list we computed the fraction of gold standard genes in the top 10% and normalized the score to 1. Using this scheme we assigned the weights 0.6, 0.3, and 0.1 to the Luo, Wang, and Steckel screens, respectively. We ran the Rank-Aggreg method 100 times, because it uses heuristics for finding the optimal ranking and therefore varies between runs.

### Rankings with RSA and riger

Besides the standard method used above, RSA (Redundant siRNA Activity) and RIGER (RNAi Gene Enrichment Ranking) [19, 20] are two established and widely used ranking methods. While the standard method is applicable to all screens, RIGER and RSA require independent measurements for multiple siRNAs per gene and can therefore only be applied to Luo and Wang. RSA is considered the most robust gene ranking method in this context [17].

RIGER and RSA methods return a *p*-value for each gene, after evaluating the contribution of the different shRNAs that target it. The RIGER algorithm calculates a *p*-value for each gene, indicative of the significance of the differential *KRAS* mutant and *KRAS* wild type cell viability caused by the gene's knock down. To this aim a weighted sum approach based on the signal to noise ratio was utilized. For the RIGER method, a simple criteria of *p*-value < 0.05 was sufficient. RSA on the other hand assigns a value to each experimental well. The inputs provided to RSA are the summarized mutant-wild type values per well for Luo and for Wang screens. According to RSA, a lower bound LB and an upper bound UB are defined, between which hit genes are sought. These bounds intuitively correspond to fold changes. Wells with lower scores than LB are guaranteed hits, whereas wells with larger scores than UB are guaranteed non-hits. After correspondence with the methods’ developers and matching of the LB and UB bounds with fold change, we opted for LB = −2 and U B = 0. There was increased confidence in favor of their being hits if the log transformed and normalized fold change is < −2. The ones having a positive fold change (> 0) correspond to the surviving cells, thus they were rejected. The most significant hits were selected based on two criteria:

The *p*-value for each gene (*p*-values are the same for all wells corresponding to a gene) should be < 0.05, and The gene should have at least two active wells (OP I _Rank < 99999 for at least two wells). This is interpreted as: at least two shRNAs below threshold.

It has to be noticed that the rank of at least two wells, having *p*-value < 0.05, should be a real number (RSA returns infinite if a rank cannot be calculated).

### Standard ranking, RSA, and RIGER broadly agree and correlate between 60 to 80%

**Figure 3:**
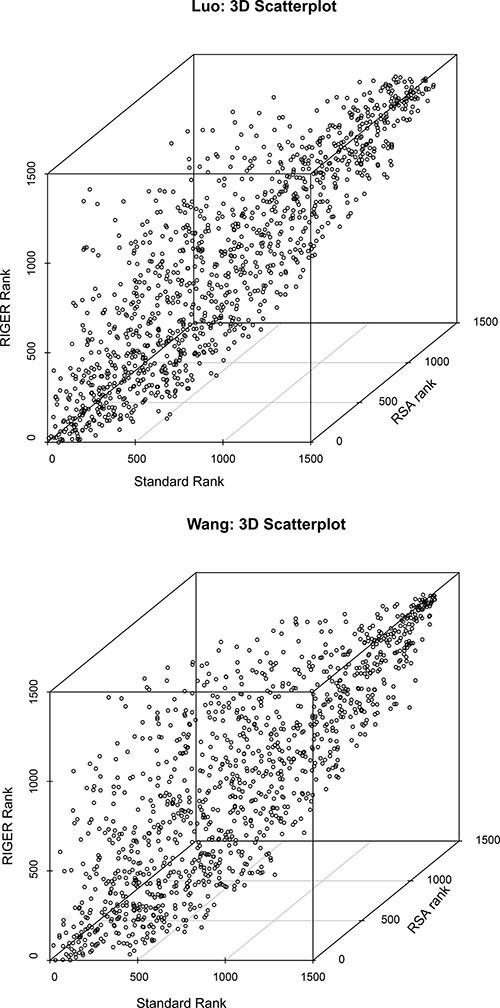
Comparison of standard (x-axis), RSA (y-axis), and RIGER (z-axis) ranking for the Luo (left) and Wang (right) screens Rankings differ, but agree broadly with correlation coefficients from 0.59 to 0.8.

Rankings with the standard method and RSA and RIGER differ, but they still correlate very well. For the Luo and Wang screen, the ranks of the three methods were mapped on three axes in a 3D scatter plot (see Figure 3). The pairwise Spearman correlations range from 0.59 to 0.8. Specifically, the are for the Luo screen: Standard-RIGER = 0.74, Standard-RSA = 0.7, RIGER-RSA = 0.8 and for the Wang screen: Standard-RIGER = 0.65, Standard-RSA =0.59, RIGER-RSA = 0.76.

### Rank aggregation and RIGER and RSA identify COPB2 as candidate SLP

The above analysis shows that standard method, RSA, and RIGER are not inconsistent and that there is not one method that can provide the one and only true ranking. At the same time, they are not in perfect agreement. To reflect this situation we consider the rank aggregation for all three screens as described above together with RIGER and RSA for the two screens of Luo and Wang. This is summarized as step 4 in Figure 1.

Table 2 shows the top three genes for this integrated ranking: coatomer protein complex, subunit beta 2 (*COPB2*), sprouty homolog 1, antagonist of FGF signaling (*SPRY1*), and nuclear receptor corepressor 1 (*NCOR1*). These three genes ranked consistently among the top 10% of all RankAggreg runs, with *COPB2* ranking 98 times, *NCOR1* 100 and *SPRY1* 88 times among the top 10%. Further, these genes were the only ones among the top 10% ranks of RankAggreg that also ranked high according to RIGER and RSA. The functionalities of all three genes were explored and associated with the nine ’hallmarks of cancer’: the nine properties that are shared among cancer cells, and are necessary for tumor initiation and expansion [21]. The function of *NCOR1* relates to the ’Resisting Cell Death’ property. The functions of *SPRY1* and *COPB2* are associated with the ’Sustaining Proliferative Signaling’ property. Information on the genes’ function was collected from GeneCards and Pubmed (www.ncbi.nlm.nih.gov/pubmed/) and Gene Cards (www.genecards.org) [22]. Silencing of *SPRY1* has been found to trigger complete regression of RAS mutant cells in the human childhood rhabdomyosarcoma (RMS) [23]. *COPB2* ranked best when the cumulative rank across all methods was considered, thus we focused on *COPB2* in our experimental validations.

**Table 2:** Hit gene ranks

Gene	Sum	RankAggreg	RIGER		RSA	
			Luo	Wang	Luo	Wang
*COPB2*	300	18	223	5	43	1
*SPRY1*	431	49	266	12	84	20
*NCOR1*	957	2	527	188	118	124

The ranks of *COPB2*, *NCOR1* and *SPRY1* by all methods. RIGER and RSA can only be calculated for the Luo and Wang screens. In this case, the ranks range from 1 to 1423, which is the number of common genes between Luo and Wang screens. RankAggreg ranks range from 1 to 1069.

We examined the expression levels of *KRAS*, *COPB2*, *NCOR1* and *SPRY1* across the five colorectal adenocarcinoma cell lines available in TCGA's cBioPortal (www.cbioportal.org) [24, 25] (Supplementary Figure 2). The expressions of *COPB2* and *NCOR1* have a much higher median than the *KRAS* expressions but wider standard deviation. This adds to our finding that they are potential *KRAS* synthetic lethal partners; their high expressions may sustain oncogenic *KRAS* signaling and knocking them down can have a big effect in cancer cell lines’ viability. On the other hand, *SPRY1* has in average lower expressions than *KRAS*.

We further examined the mutation status of KRAS and its three candidate synthetic lethal partners in published colorectal and pancreatic cancer cell lines [24, 25]. We are expecting that *KRAS* is very rarely mutated together with the three genes, which sustains the cell line's viability. The results shown in Table 3 prove that this hypothesis holds.

**Table 3:** Co-occurring mutations of the hit genes across publicly available pancreatic and colorectal cancer cell lines

Study	Samples	% *KRAS*	% *COPB2*	% *NCOR1*	% *SPRY1*
DFCI, Cell Reports 2016 [26]	619	28%	2.3%	2.9%	0.5%
Genentech, Nature 2012 [27]	72	51%	4%	4%	1.4%
MSKCC, Genome Biol 2014 [28]	138	55%	0	0	0
TCGA, Nature 2012 [29]	276	42%	2.20%	4%	1.3%
Colorectal Adenocarcinoma, TCGA Provisional [30]	633	43%	2.2%	4%	1.3%
ICGC, Nature 2012 [31] UTSW,	99	95%	0	0	0
Nat Commun 2015 [32] QCMG,	109	93%	2.8%	6%	6%
Nature 2016 [33] UTSW,	383	90%	0.3%	0.5%	0
Nat Commun 2015 [32] Pancreatic	109	93%	2.8%	6%	6%
Adenocarcinoma, TCGA Provisional [30]	149	91%	0.7%	2.7%	0

### Cell lines for COPB2 knock-down

To validate the above finding, we knocked down *COPB2* in the human pancreatic cancer cell lines shown in Table 4. We chose these cell lines, because they are well-known standard cells, easy to handle, suitable for transfection experiments and have different genotypes. We examined the effects of *COPB2* knock down on selected protein levels to find a link between *COPB2*, fewer living cells, and autophagic cell death.

**Table 4:** Experimental cell lines

Cell line	*KRAS* status
MIAPaCa2	*KRAS*^G12C/G12C^ mutant
Hs766T	*KRAS*^Q61H/Q61H^ mutant
Panc1	*KRAS*^+/G12D^ mutant
BxPC3	*KRAS*^+/+^ wild type
PaCaDD165	*KRAS*^+/+^ wild type
DLD-1	*KRAS*^+/G13D^ mutant

**Figure 4:**
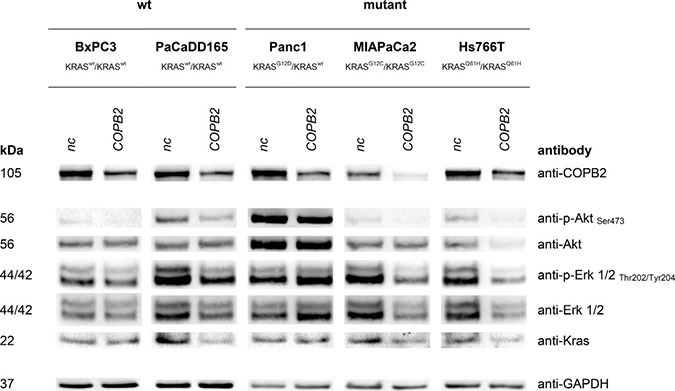
KRAS signaling: Top western blot row shows that *COPB2* is effectively knocked down in all cell lines The rest rows show the effects of *COPB2* knock down on downstream KRAS signaling. GAPDH is used as control.

**Figure 5:**
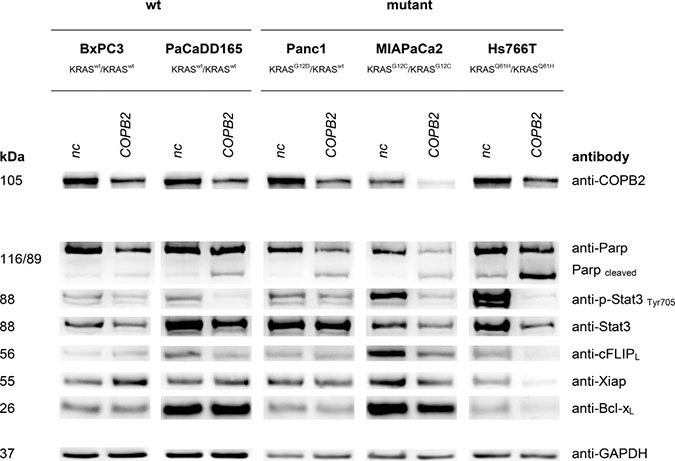
Apoptosis: Top western blot row shows that *COPB2* is effectively knocked down in all cell lines The rest show the effects of *COPB2* knock down on apoptosis signaling. GAPDH is used as control.

**Figure 6:**
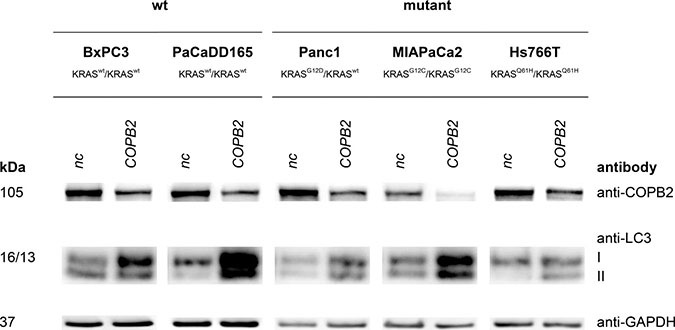
Autophagy induction: Top western blot row shows that *COPB2* is effectively knocked down in all cell lines The rest show the effects of *COPB2* knock down on down on autophagy-related proteins. GAPDH is used as control.

Western Blot analysis (see Figures 4, 5, 6) revealed effects of *COPB2* knock-down on the cellular level of proteins involved in KRAS signaling, apoptosis and autophagy in all pancreatic tested cell lines. These figures are duplicated from the original for presentation and explanation purposes. The original western blot is provided in Supplementary Figure 1.

### *COPB2* knock down and signalling

We first examined the phosphorylation of downstream effectors of KRAS, Protein kinase B (Akt) and extracellular-signal-regulated kinases ERK1/2, in order to see if the downstream KRAS pathway was silenced. Indeed, the phosphorylation levels of the selected proteins were lower after *COPB2* knock-down only in MIAPaCa2 and Hs766T cells (homozygous *KRAS* mutant).

### *COPB2* knock down and apoptosis

We then examined how cell apoptosis is affected by *COPB2* knock down. A good indicator for apoptosis induction is Poly (ADP-Ribose) Polymerase (PARP) cleavage. Enhanced levels of cleaved PARP were detected after *COPB2* knockdown in all cell lines compared to their negative control, except *KRAS* wild type BxPC3. Therefore, apoptosis, which could contribute to the reduction of living cells, was induced in those cell lines. Anti-apoptotic proteins like cFLIP, XIAP and BCL-XL showed a decreased expression after *COPB2* knock-down in Hs766T cells. Importantly, Signal Transducer and Activator of Transcription 3 (STAT3), a key transcription factor of cell growth and apoptosis, showed a reduction of its phosphorylated form in cell lines MIAPaCa2, Hs766T and PaCaDD165, meaning that downstream pro-survival functions might be inhibited.

### *COPB2* knock down and autophagy

Finally, conversion of Microtubule-associated protein light chain 3 I (LC3) to LC3 II is a marker of autophagy with the amount of LC3 II negatively correlating to the number of autophagosomes. In our comparison of the negative control to the *COPB2* knock-down there was a clear increase of LC3II level in Hs766T, PaCaDD165, Panc1 and MIAPaCa2, thereby exhibiting an increase in autophagy after *COPB2* knock-down.

### *COPB2* knock down and living cell counts

**Figure 7:**
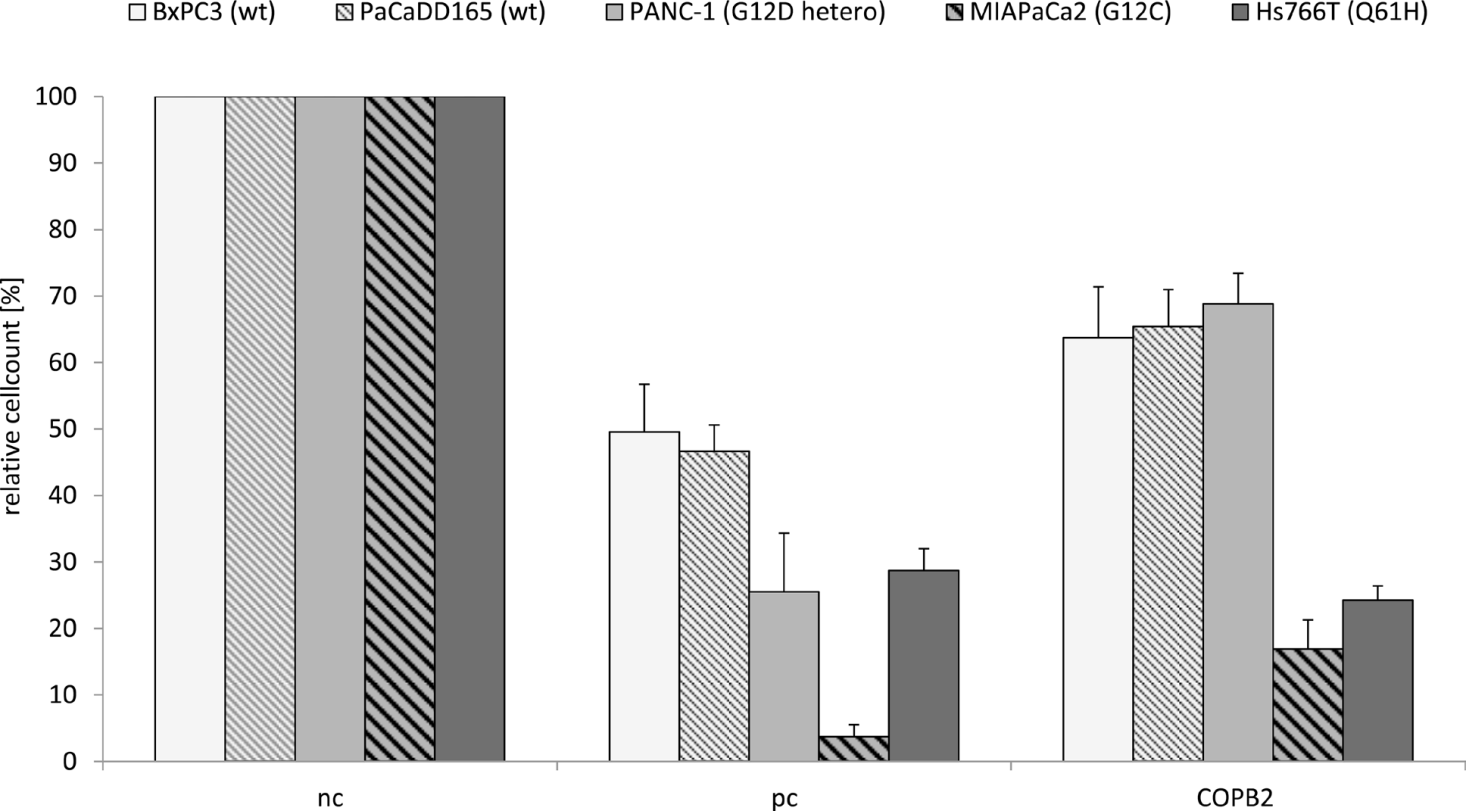
*COPB2* knock down reduces number of living cells in pancreatic cancer cell lines depending on their *KRAS* genotype Each cell line's *KRAS* genotype is shown in Table 4. After 72 h of transfection using 72 nM non-coding siRNA (negative control, nc), *KIF11* siRNA (positive control, pc) and *COPB2* siRNA (*COPB2*) cell number was quantified in *KRAS* wildtype (wt) and mutated pancreatic cancer cell lines. The *p*−value is *p* < 0.01 in cell line specific comparison.

**Figure 8:**
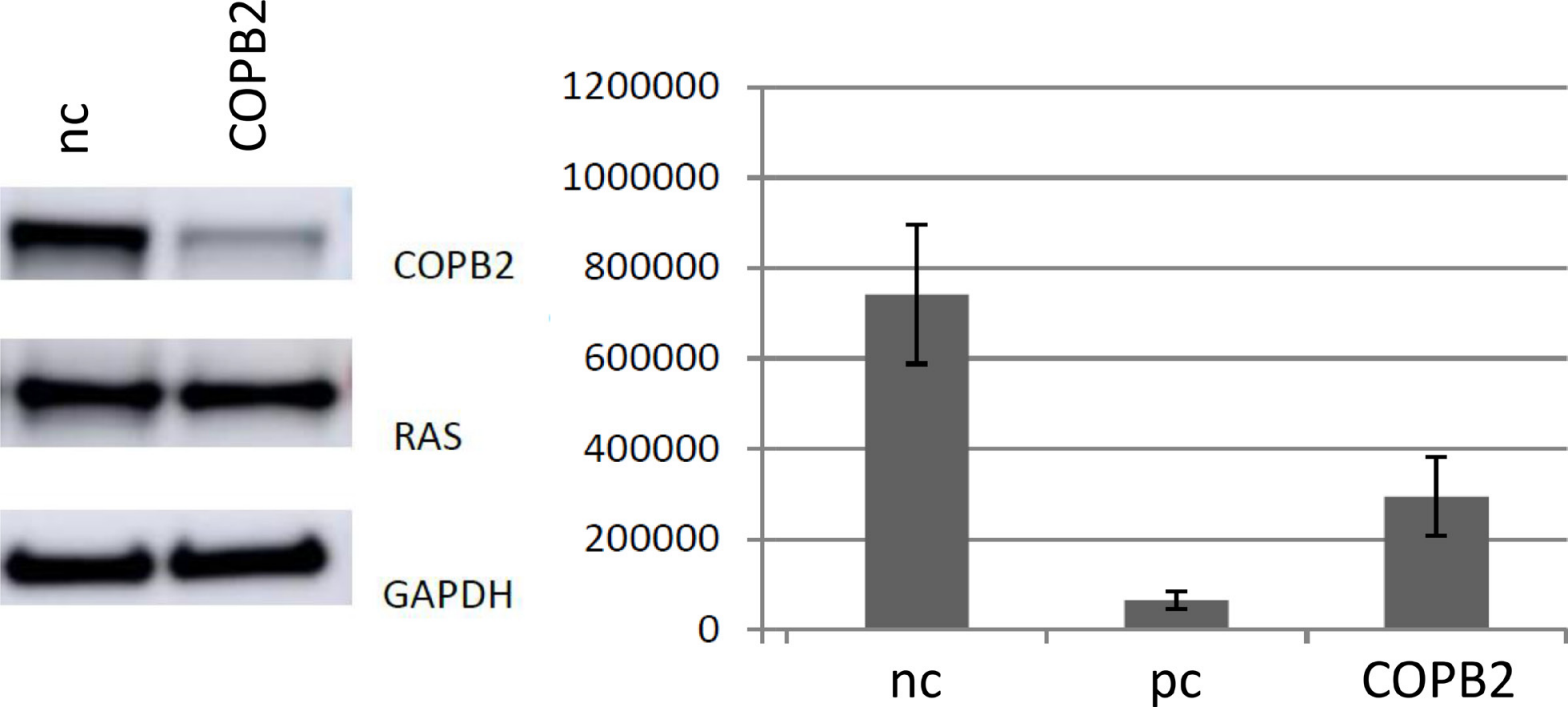
*Left* Knockdown of *COPB2* in DLD-1 cells, indicating a knock-down of *COPB2*, but no significant loss of *KRAS* expression in this cell line *Right* Knockdown of *COPB2* in DLD-1 cells leads to a reduction in cell number after 72 h of transfection (*p* < 0.01). nc stands for negative control, pc stands for positive controltion of the gene of interest.

More importantly, the change in expression levels of relevant genes is complemented by changes in living cell counts (see Figures 7 and 8) Homozygous pancreatic *KRAS* mutated cell lines MIAPaCa2 and Hs766T, show a high reduction of living cells after *COPB2* knock down onto 16.93% (± 4.34%) and 24.22% (± 2.19%) compared to the negative control (nc). The number of living cells in the *KRAS* wild type cell lines BxPC3 and PaCaDD165, was reduced to 63.74% (± 7.64%) and 65.44% (± 5.53%). Heterozygous *KRAS* mutated cell line Panc1 also had a cell count of 68.81% (± 4.62%). These results show that there is a significant difference regarding the reduction of living cells after *COPB2* knock down in homozygous *KRAS* mutated cell lines, MIAPaCa2 and Hs766T, compared to the *KRAS* wildtype cell lines, BxPC3 and PaCaDD165, and *KRAS* heterozygous mutated Panc1.

In addition, there is a significant difference regarding the reduction of living cells after *COPB2* knock down in the heterozygous colorectal cancer cell line DLD-1, compared to negative control (Figure 8).

The above results confirm our speculation about a correlation between *KRAS* mutation status and *COPB2* sensitivity on the selected pancreatic and colorectal carcinoma cell lines.

Single inhibition of *KRAS* or simultaneous inhibition of both *KRAS* and *COPB2*, to further support our finding, has not been conducted. However, we recently performed a similar experiment where we inhibited *KRAS* and the anti-apoptotic genes *BCLXL, FLIP, MCL1L, SURVIVIN* and *XIAP* in a panel of murine and human pancreatic cancer cell lines, including the ones that we employed in the current work. Single inhibition of *KRAS* had little effect in cell apoptosis. Simultaneous inhibition of *KRAS* and the anti-apoptotic proteins significantly induced apoptosis and inhibited proliferation of the cell. This finding was validated *in vivo* on mice. Given the role of *COPB2* in apoptosis signaling, as shown by our experiments (see Figure 5), we suspect that the simultaneous inhibition of *COPB2* and *KRAS* in pancreatic cancer cells will be more effective than *COPB2* silencing alone.

## DISCUSSION

In this work, we analyzed three different RNAi screens that are designed to detect KRAS synthetic lethal partners and combined them using a computational framework. Our methodology detected *COPB2* as the most promising *KRAS* synthetic lethal partner, which was further experimentally evaluated.

Our experimental data, comparing pancreatic and colorectal cancer cell lines with different *KRAS* genotypes after *COPB2* knock down, implicate that this knock down has a higher negative impact on the number of living cells in the case of homozygous pancreatic *KRAS* mutated cells and heterozygous colorectal cells, with respect to the *KRAS* wild type cells (Figures 7 and 8).

Knock down approaches and western blot analysis in pancreatic cell lines demonstrated that *COPB2* influences KRAS signaling, apoptosis and autophagy (see Figures 4, 5, and 6). Pancreatic tumor cells depend on KRAS signaling, on evasion of apoptosis and on autophagy to sustain themselves and proliferate [34], [21], [35]. Thus, when the above are impaired, pancreatic tumor cells are put under stress and are more susceptible to death. Regarding autophagy, we noticed an increase in LC3 protein after *COPB2* knock down (Figure 6), which is interpreted as a decrease in autophagy [36]. In the past, it has been shown that *COPB2* (COPI subunit) knock down disables autophagy [37], which in turn leads to abortive autophagy [38]. With respect to pancreatic cancer, it is known that pancreatic tumor growth relies on autophagy [35], especially in *KRAS* mutated cells where metabolism is deprogrammed. Thus when autophagy is decreased, tumor growth is inhibited. Further investigations could involve measurement of the exact amount of apoptotic cells, the cause of cell death, qPCR to determine the effects on mRNA level, migration assays and proliferation assays.

Knock down approaches and western blot analysis in colorectal cell line DLD-1 demonstrated that *COPB2* was efficiently knocked-down whereas *KRAS* remained intact. This knock-down resulted in significantly reduced number of colorectal cancer living cells (Figure 8). Although we have not tested the levels of LC3 protein after *COPB2* knock-down in DLD-1 cells, we suspect that autophagy is one of their survival mechanisms too, based on existing literature [39, 40].

Expression of *COPB2* maybe linked to KRAS signaling as follows: Activated KRAS activates downstream RalGDS signaling [41]. As a consequence, the small GTPases RALA and *RALB* are activated. They play a major role in vesicular trafficking and tumor proliferation [42]. It is noticeable that *RALB* also promotes autophagy. In mammals, the coatomer sub-unit, which contains *COPB2*, can only be recruited when activated small guanine triphosphatases (GTPases) attract coat proteins to specific membrane export sites, thereby linking coatomers to export cargos [43]. We believe that the involvement of *RALA* and *RALB* in vesicular trafficking, in which *COPB2* is also involved, and the co-localization of *KRAS* transcripts in the Golgi complex [44] may support the positive feedback loop: GTP *KRAS* → *COPB2* → GTP *KRAS*.

We note here that, according to Ihle et al., the Ral-GDS pathway is enabled only when the activated *KRAS*^G12C^ binds to it [45]. *KRAS*^G12D^, on the other hand, activates PI3K signaling. In our experiment, Panc1 heterozygous mutant *KRAS*^G12D^ cell line exhibits similar behavior with wild type *KRAS* pancreatic tumor cells. We believe that in this case the PI3K pathway is activated instead of the Ral-GDS. In contrast, *COPB2* knock-down significantly decreases the viability of the homozygous *KRAS*^G12C^ MIAPaCa2 cell line, probably due to the activation of Ral-GDS. Another possible reason for the behavior of Panc1, is that Panc1 has high latency period before tumor progression, comparable to that of the wild type BxPC3 cell line [46]. So maybe the behavior of Panc1 after 72 h of treatment, which we examined, is closer to wild type cells. In regards to why this happens, it seems that the fate of a cell that contains both oncogenic and wild type *KRAS*, depends on the type of the wild type allele (*RAS* -GDP or *RAS* -GTP) and on the status of other tumor suppressor and cell cycle inhibitor genes [47].

The current findings agree with past studies, as there is supportive evidence from literature that *COPB2* knock down undermines survival of *KRAS* mutant cells, meaning that survival of cells is decreased by the knock down; experiments on non small cell lung cancer (NSCLC) cells show that there is a COPI addiction in *KRAS* and *LKB1* mutated cell lines [48].

As a last note, we used RNAi knock-down technology to silence the gene of interest because the original screens, from which we derive our hypotheses, use RNAi. For the sake of comparability we decided for RNAi and not CRISPR/Cas9 which is another promising gene-editing technology. Unniyampurath et al. explain that RNAi and CRISPR/Cas9 are two different technologies, of which the outcome does not necessarily overlap [49]. This mainly happens because CRISPRs regulate gene transcription by altering the DNA (pre-transcription level) whereas RNAi regulates gene transcription by acting posttranscriptionally. siRNA and shRNA approaches are excellent as the first attempt to understand the function of the gene of interest. CRISPR/Cas9 system is the next logical step to fully elucidate the function of the gene and to validate phenotypes observed by knock-down approaches.

This study has shown that consistent data integration identified a novel synthetic lethal partner of *KRAS*, which could be experimentally confirmed in our knock-down experiments. However, future work has to be done to validate our finding *in vivo* and *in vitro*, with additional knock-down experiments with other RNAi constructs and more cell lines.

## MATERIALS AND METHODS

### Standard RNAi screen ranking method

Luo and Wang screens contain three replicate measurements for each cell line. Having only three replicates per cell line, normal distribution was safely assumed. All the shRNAs targeting a gene were included and ranked based on their differential effect on the viability of the *KRAS* mutant versus *KRAS* wild type cells. This was captured by the paired *t*-test. The null hypothesis tested was that depletion of a specific shRNA has no significant effect on the viability between *KRAS* mutant and *KRAS* wild type cell lines. The respective alternative hypothesis was that the viability of the *KRAS* mutant cells decreases more than the viability of their isogenic wild type counterparts. A gene was reported as hit when the best-ranking of all the shRNAs that target it was below threshold. Our assumption at this point is that if one shRNA depletes the target gene, then the observed phenotype is cell death. Regarding the Steckel screen, no replicate data were provided. Thus, only the difference of the z-scores (mutant-wild type) was considered. This is an apoptosis screen, so the result has been inverted in order to be comparable to the other two viability screens. The rank r in this case was calculated as:

$$\text{r}=-\text{rank}(\text{z}{.\text{score}}_{\text{mut}}-\text{z}{.\text{score}}_{\text{wt}})$$

The *p*-value threshold for the Standard method and for the Luo and Wang screens was set to 0.05. The z-score threshold for the standard method and for the Steckel screen was set to -1.

### Rank aggregation

We aggregated the common gene rankings from the individual screens to a global ranking, which best reflects the ordering of the input lists. Rank aggregation is an optimization problem and one possible way to generate the final list is by using heuristic functions. To this aim, the RankAggreg R method was used, which supports the Genetic Algorithm (GA) and Cross Entropy Monte Carlo (CE) heuristics [18].

Both heuristics are iterative. The methods converge when the optimal super-list remains optimal for a number of consecutive iterations, based on the distance between each iteration's output list with each of the input lists. This distance has to be as small as possible. Spearman's footrule distance was chosen because it can quickly be computed in linear time and is a non-trivial 2-approximation of Kemeny's local optimization, as proven by the Diakonis-Graham inequality: For any two full lists σ, τ: K (σ, τ) ≤ F (σ, τ ) ≤ 2K (σ, τ ), where K is the Kemeny local optimization and F the Spearman footrule distance [50].

### Spearman's footrule distance

We want to compare an aggregated ranking r against m rankings r_1,..._, r_m_. All of the rankings range from 1 to n. We use Spearman's footrule to compare the displacement between r and r_k_ and then we sum up these displacements for all m rankings. Formally, the displacement D between r and r_k_ is defined as

$${\text{D}}_{\text{r}}(\sigma_{\text{k}})=\sum_{\text{i}=1}^{\text{n}}\left|\sigma_{\text{k}}(\text{i})-\text{i}\right|$$ 1

where σ_k_ is a permutation of n elements, such that the element at position i in the ranking r is at position σ_k_ (i) in the ranking r_k_. I.e. Spearman's footrule sums up the differences in position in r and r_k_ for all elements i. Note, if the rankings r and rk are identical, then D_r_ (σ_k_ ) = 0.

To evaluate the distance of a ranking r to r1, …, rm we simply sum up:

$${\text{D}}_{\text{r}}=\sum_{\text{k}=1}^{\text{m}}{\text{D}}_{\text{r}}\left(\sigma_{\text{k}}\right)$$ 2

Now we can optimize. For rank aggregation we search for a ranking r, that minimizes D_r_. This minimization step is implemented in the RankAggreg algorithm [18].

### COPB2 knock down

### Cell culture

The human pancreatic cancer cell lines DLD-1, BxPC3, MIAPaCa2 and Panc1 were obtained from the American Type Culture Collection. Hs766T was kindly provided by Tatjana Crnogorac-Jurcevic (Centre for Molecular Oncology, Barts Cancer Institute, UK). The primary cell line PaCaDD165 was established following the Dresden Outgrowth protocol [51].

DLD-1, BxPC3 and Panc1 cells were cultivated in RPMI-1640 supplemented with 10% fetal calf serum (FCS), MIAPaCa2 cells in DMEM with 10% FCS and 2.5% horse serum and Hs766T in DMEM with 10% FCS. Primary cells were cultured in DMEM with 20% FCS and 50% K-SFM. All cell lines were maintained in a humidified 5% C O2 incubator at 37^°^C.

### Transfection

For each transfection, 72 nM siRNA were transfected using OligofectamineTM Transfection Reagent (Invitrogen) according to the manufacturer's protocol. Transfected cells were analyzed after 72 h. SiRNA against *COPB2* was acquired from QIAGEN (FlexiTube siRNA Cat. No. 1027415) with the following target sequence: CAGGTTTCAAGGGTAGTGAAA. Noncoding siRNA Allstars provided by QIAGEN was used as a negative control. SiRNA against essential cytoskeletal motor protein coding gene *KIF11* with the sequence AACUGAAGACCUGAAGACAAU served as a positive control. Gene silencing was confirmed by Western Blot analysis.

### Western blot analysis

Transfected cells were washed and homogenized in a protein lysis buffer (RIPA buffer). Protein concentrations were quantified using BCA Protein Assay Kit (Pierce). 10 μg of each sample were denaturized at 95^°^C for 10 min and further processed using the NuPAGE SDSPAGE Gel System (Invitrogen). Proteins were transferred electrophoretically to a nitrocellulose membrane (Hybond ECL, GE Healthcare), which was treated afterwards with 5% skimmed milk powder in TBS Tween 0.1%. Primary antibodies were applied to the membrane according to manufactures’ protocol: anti-p-AKT Ser473 (Cell Signaling), anti-AKT (Cell Signaling), anti-BCL-X S/L (Santa Cruz Biotechnology), anti-cFLIP S/L (Adipogen) anti-COPB2 (novus Biologicals), anti-p-ERK1/2 Thr202/Tyr204 (Cell Signaling), anti-ERK1/2 (Cell Signaling), anti-GAPDH (Cell Signaling), anti-KRAS (Santa Cruz Biotechnology), anti-LC3 (Cell Signaling), anti-pSTAT3 (Tyr705) (Cell Signaling), anti-STAT3 (Cell Signaling), anti-PARP (Cell Signaling), anti-XIAP (BD Biosciences). The membrane was washed and incubated with the appropriate horseradish peroxidase-conjugated antiMouse or anti-Rabbit secondary antibodies (Cell Signaling). The chemiluminiscent reaction was initialized using Immobilon Western Chemiluminescent HRP Substrate (Millipore) and detected by G:Box Chemi XT4 (Syngene) (Figures 4, 5, 6).

### Quantification of living cells

A reduction of living cells can refer to proliferation inhibition as well as apoptosis induction. Transfected cells were trypsinized following standard cell culture practices and counted by TC-20 Automated Cell Counter (BIORAD). Count of *COPB2* siRNA treated cells (*COPB2*) were normalized to its noncoding siRNA control (nc).

## CONCLUSIONS

In this work we aim at the detection of reliable and robust *KRAS* Synthetic Lethal Partners, though combination of exiting datasets. In summary, we have shown that a meta analysis consistently integrating large scale RNAi data is able to generate new hypotheses, which were not prominent in the individual screens. Here, we identified *COPB2* as such candidate that was screened three times and missed three times. However, in the meta analysis, *COPB2* ranks top and experimental validation confirms its role as synthetic lethal partner for *KRAS*. Evidence from literature and *COPB2*'s role in autophagy and apoptosis further support the experimental finding.

## SUPPLEMENTARY MATERIALS FIGURES AND TABLES




