# Supplementary material for: Detection of COPB2 as a KRAS synthetic lethal partner through integration of functional genomics screens
Source: Oncotarget. 2017 Mar 10;8(21):34283–97. doi: 10.18632/oncotarget.16079 (PMC5470967; doi:10.18632/oncotarget.16079)
Supplement: Supplementary file 1 [file oncotarget-08-34283-s001.pdf]

Detection of COPB2 as a KRAS synthetic lethal partner through integration of functional genomics screens

Supplementary Materials

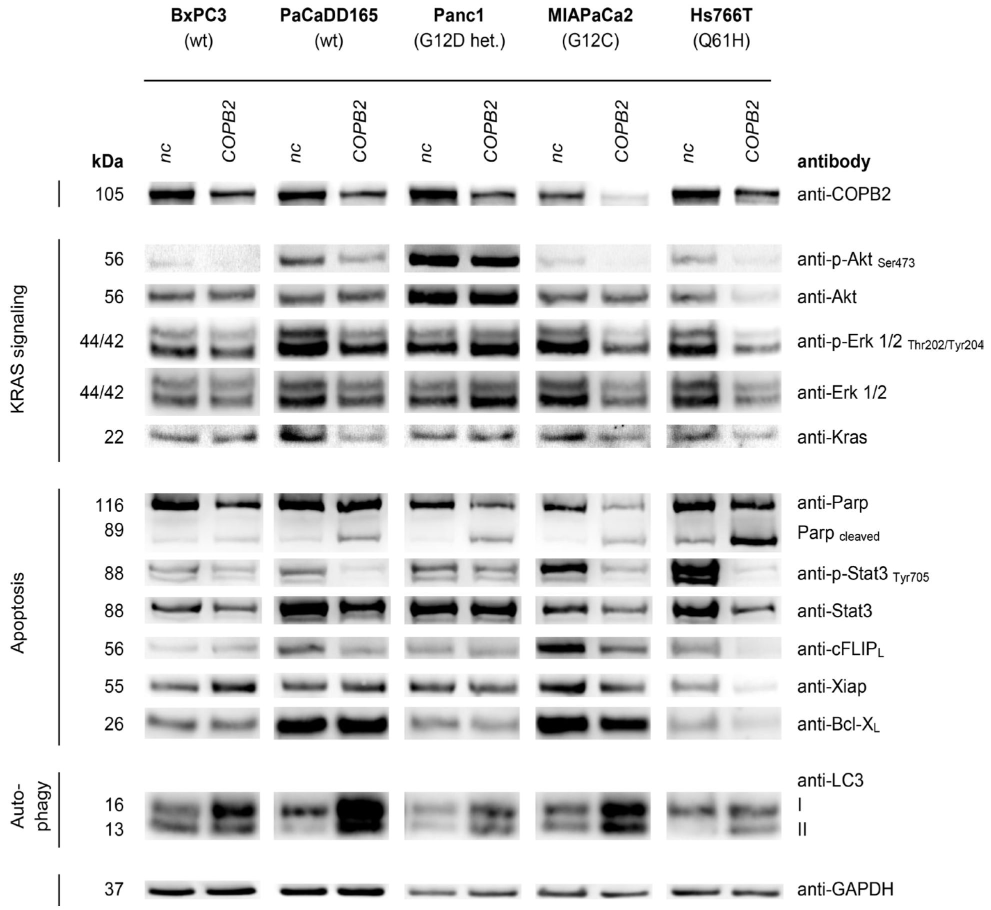

Supplementary Figure 1: Western Blot analysis reveals influence of *COPB2* knockdown on expression of cellular proteins and transcription factors involved in apoptosis, autophagy induced cell death and KRAS signaling

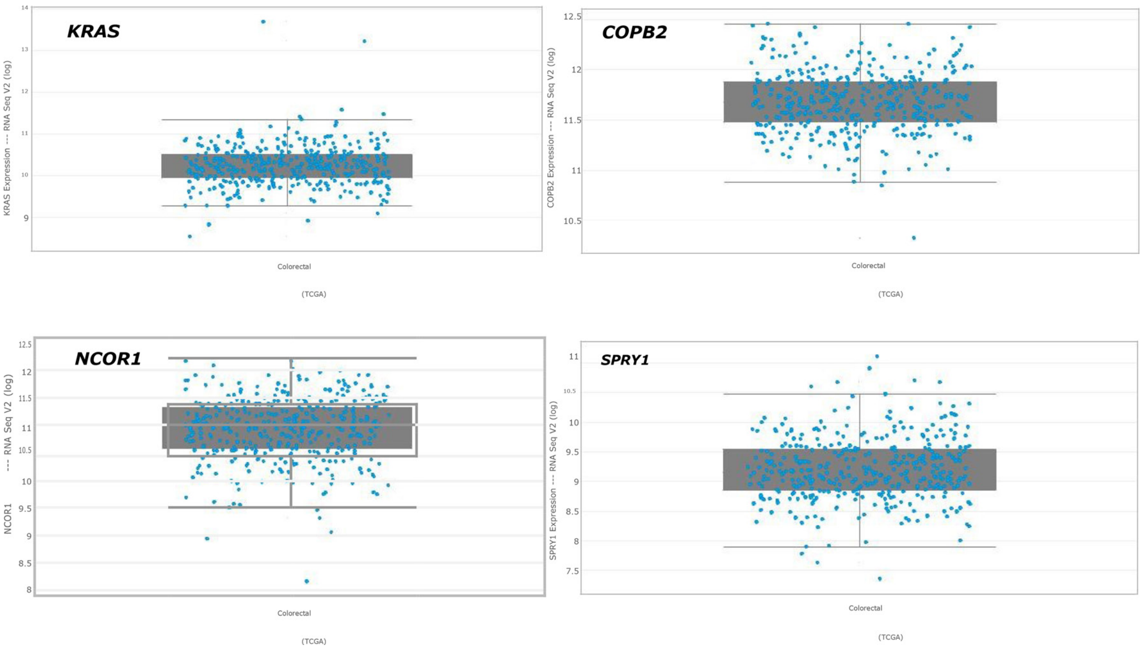

Supplementary Figure 2: Expression levels of *KRAS, COPB2*, *NCOR1* and *SPRY1* across the five colorectal adenocarcinoma cell lines available in TCGA's cBioPortal (www.cbioportal.org) [30, 31]. The expressions of COPB2 and NCOR1 have a much higher median than the KRAS expressions but wider standard deviation. SPRY1 has in average lower expressions than KRAS

SUPPLEMENTARY MATERIALS

KRAS SLPs in literature or 'gold standard genes' (GSGs)

In order to retrieve the *KRAS* SLPs we searched PubMed, initially with the query "*KRAS* synthetic lethal". The result of the query, as of 17-03-2014, contained 28 papers, including *KRAS* SLPs in species different from human. Some of the retrieved studies detected drug *di* and gene *gi* combinations as *KRAS* SLPs [1]. This means that a gene *gi* is SLP of *KRAS* only under the presence of a specific drug *di*. To avoid missing any potential SLPs, the query was expanded to "RAS synthetic lethal". This resulted in 68 papers, including *KRAS* and other members of the Ras-family (*HRAS, NRAS*) SLPs as well.

Supplementary Table 1: KRAS SLPs in literature. See Supplementary_Table_1

REFERENCES

1. Corcoran RB, Cheng Ka, Hata AN, Faber AC, Ebi H, Coffee EM, Greninger P, Brown RD, Godfrey JT, Cohoon TJ, Song Y, Lifshits E, Hung KE, et al. Synthetic lethal interaction of combined BCL-XL and MEK inhibition promotes tumor regressions in KRAS mutant cancer models. Cancer cell. 2013; 23:121–8.
2. Bennardo N, Cheng A, Huang N, Stark JM. Alternative-NHEJ is a mechanistically distinct pathway of mammalian chromosome break repair. PLoS genetics. 2008; 4.
3. Luo J, Emanuele MJ, Li D, Creighton CJ, Schlabach MR, Westbrook TF, Wong Kk and Elledge SJ. A genome-wide RNAi screen identifies multiple synthetic lethal interactions with the Ras oncogene. Cell. 2009; 137:835–848.
4. Steckel M, Molina-Arcas M, Weigelt B, Marani M, Warne PH, Kuznetsov H, Kelly G, Saunders B, Howell M, Downward J, Hancock DC. De-termination of synthetic lethal interactions in KRAS oncogene-dependent cancer cells reveals novel therapeutic targeting strategies. Cell research. 2012; 22:1227–45.
5. Hesketh R. Introduction to Cancer Biology. Cambridge University Press. 2012.
6. Gilad O, Nabet BY, Ragland RL, Schoppy DW, Smith KD, Durham AC, Brown EJ. Combining ATR suppression with oncogenic Ras synergistically increases genomic instability, causing synthetic lethality or tumorigenesis in a dosage-dependent manner. Cancer research. 2010; 70:9693–702.
7. Sale MJ, Cook SJ. The BH3 mimetic ABT-263 synergizes with the MEK1/2 inhibitor selumetinib/AZD6244 to promote BIM-dependent tumor cell death and inhibit acquired resistance. The Biochemical journal. 2013; 450:285–94.
8. Barbie Da, Tamayo P, Boehm JS, Kim SY, Moody SE, Dunn IF, Schinzel AC, Sandy P, Meylan E, Scholl C, Fröhling S, Chan EM, Sos ML et al. Systematic RNA interference reveals that oncogenic KRAS-driven cancers require TBK1. Nature. 2009; 462:108–12.
9. Sarthy AV, Morgan-Lappe SE, Zakula D, Vernetti L, Schurdak M, Packer JCL, Anderson MG, Shirasawa S, Sasazuki T, Fesik SW. Survivin depletion preferentially reduces the survival of activated K-Ras-transformed cells. Molecular cancer therapeutics. 2007; 6:269–76.
10. Ho J, Bretscher A. Ras regulates the polarity of the yeast actin cytoskeleton through the stress response pathway. Molecular biology of the cell. 2001; 12:1541–55.
11. Puyol M, Martin A, Dubus P, Mulero F, Pizcueta P, Khan G, Guerra C, Santamaria D, Barbacid M. A synthetic lethal interaction between K-Ras oncogenes and Cdk4 unveils a therapeutic strategy for non-small cell lung carcinoma. Cancer cell. 2010; 18:63–73.
12. Hattori H, Skoulidis F, Russell P, Venkitaraman R. Context Dependence of Checkpoint Kinase 1 as a Therapeutic Target for Pancreatic Cancers Deficient in the BRCA2 Tumor Suppressor. Molecular Cancer Therapeutics. 2011; 10:670–678.
13. Ramdzan ZM, Vadnais C, Pal R, Vandal G, Cadieux C, Leduy L, Davoudi S, Hulea L, Yao L, Karnezis AN, Paquet M, Dankort D, Nepveu A. RAS Transformation Requires CUX1-Dependent Repair of Oxidative DNA Damage. PLoS biology. 2014; 12.
14. Wu X, Lippman SM. An intermittent approach for cancer chemoprevention. Nature reviews. Cancer. 2011; 11:879–85.
15. Meyskens FL, Gerner EW. Back to the future: mechanism-based, mutation-specific combination chemoprevention with a synthetic lethality approach. Cancer prevention research (Philadelphia, Pa.). 2011; 4:628–32.
16. Kumar MS, Hancock DC, Molina-Arcas M, Steckel M, East P, Diefenbacher M, Armenteros-Monterroso E, Lassailly F, Matthews N, Nye E, Stamp G, Behrens A, Downward J. The GATA2 transcriptional network is requisite for RAS oncogene-driven non-small cell lung cancer. Cell. 2012; 149:642–55.
17. Shen S, Mao CQ, Yang XZ, Du XJ, Liu Y, Zhu YH, Wang J. Cationic Lipid-Assisted Polymeric Nanoparticle Mediated GATA2 siRNA Delivery for Synthetic Lethal Therapy of KRAS Mutant Non-Small-Cell Lung Carcinoma. Molecular pharmaceutics. 2014.
18. Karachaliou N, Mayo C, Costa C, Magrí I, Gimenez-Capitan A, Molina-Vila MA, Rosell R. KRAS mutations in lung cancer. Clinical lung cancer. 2013; 14:205–14.
19. Nilsson LM, Forshell TZP, Rimpi S, Kreutzer C, Pretsch W, Bornkamm GW, Nilsson Ja. Mouse genetics suggests cell-context dependency for Myc-regulated metabolic enzymes during tumorigenesis. PLoS genetics. 2012; 8.
20. Singh A, Sweeney MF, Yu M, Burger A, Greninger P, Benes C, Haber Da and Settleman J. TAK1 inhibition promotes apoptosis in KRAS-dependent colon cancers. Cell. 2012; 148:639–50.
21. Takashima A, Faller DV. Targeting the RAS oncogene. Expert opinion on therapeutic targets. 2013; 17:507–31.
22. Shaw AT, Winslow MM, Magendantz M, Ouyang C, Dowdle J, Subramanian A, Lewis TA, Maglathin RL, Tolliday N, Jacks T. Selective killing of Kras mutant cancer cells by small molecule inducers of oxidative stress. Proceedings of the National Academy of Sciences of the United States of America. 2011; 108:8773–8.
23. Chen Z, Forman LW, Williams RM, Faller DV. Protein kinase C-delta inactivation inhibits the proliferation and survival of cancer stem cells in culture and *in vivo*. BMC cancer. 2014; 14:90.
24. Wang Y, Ngo VN, Marani M, Yang Y, Wright G, Staudt LM, Downward J. Critical role for transcriptional repressor Snail2 in transformation by oncogenic RAS in colorectal carcinoma cells. Oncogene. 2010; 29:4658–70.
25. Scholl C, Fröhling S, Dunn IF, Schinzel AC, Barbie D, Kim SY, Silver SJ, Tamayo P, Wadlow RC, Ramaswamy S, Döhner K, Bullinger L, Sandy P et al. Synthetic lethal interaction between oncogenic KRAS dependency and STK33 suppression in human cancer cells. Cell. 2009; 137:821–34.

26. Singh A, Greninger P, Rhodes D, Koopman L, Violette S, Bardeesy N, Settleman J. A gene expression signature associated with "K-Ras addiction" reveals regulators of EMT and tumor cell survival. Cancer cell. 2009; 15:489–500.

27. Tran PT, Shro EH, Burns TF, Thiyagarajan S, Das ST, Zabuawala T, Chen J, Cho YJ, Luong R, Tamayo P, Salih T, Aziz K, Adam SJ et al. Twist1 suppresses senescence programs and thereby accelerates and maintains mutant Kras-induced lung tumorigenesis. PLoS genetics. 2012; 8:e1002650.

28. Yang WS, Stockwell BR. Synthetic lethal screening identifies com-pounds activating iron-dependent, nonapoptotic cell death in oncogenic-RAS-harboring cancer cells. Chemistry & biology. 2008; 15:234–45.

29. Licciulli S, Kissil J. WT1: a weak spot in KRAS-induced transformation. The Journal of clinical investigation. 2010; 120:9–12.

30. Cerami E, Gao J, Dogrusoz U, Gross BE, Sumer SO, Aksoy BA, Jacobsen A, Byrne CJ, Heuer ML, Larsson E, Antipin Y, Reva B, Goldberg AP et al. The cBio Cancer Genomics Portal: An Open Platform for Exploring Multidimensional Cancer Genomics Data. Cancer Discov. 2012; 2.

31. Gao J, Aksoy BA, Dogrusoz U, Dresdner G, Gross B, Sumer SO, Sun Y, Jacobsen A, Sinha R, Larsson E, Cerami E, Sander C, Schultz N. Integrative Analysis of Complex Cancer Genomics and Clinical Pro les Using the cBioPortal. Sci. Signal. 2013; 6:pl1–pl1.
