# Supplementary material for: Detection of COPB2 as a KRAS synthetic lethal partner through integration of functional genomics screens
Source: Oncotarget. 2017 Mar 10;8(21):34283–97. doi: 10.18632/oncotarget.16079 (PMC5470967; doi:10.18632/oncotarget.16079)
Supplement: Supplementary file 2 [file oncotarget-08-34283-s002.docx]

Table 1: *KRAS* SLPs in literature

| *KRAS* SLPs | From datasets [***^1^***](#page1) | From rest literature |
| --- | --- | --- |
| *ALT-NHEJ* pathway [[2](#page6)] |  | X |
| *APC / C* complex (e.g *ANAPC1*, | X | X |
| *ANAPC4, CDC16, CDC27* ) [[3](#page6)-[5](#page6)] |  | X |
| *ATR* [[6](#page6)] |  |  |
| *BCL2* [[7](#page6)] |  | X |
| *BCL2L1* [[8](#page6)] |  | X |
| *BCL2L1* + MEKi [[1](#page6)] |  | X |
| *BIRC5* [[3](#page6), [9](#page6)] | X | X |
| *BRIX1* [[3](#page6)] |  | X |
| *cAMP* / PKA pathway [[10](#page6)] | X |  |
| *CASC5* [[3](#page6)] |  |  |
| *CCNA2* [[3](#page6)] | X |  |
| *CDC6* [[4](#page6)] | X |  |
| *CDCA8* [[3](#page6)] | X | X |
| *CDK4* [[11](#page6)] |  |  |
| *CHEK1* [[6](#page6), [12](#page6)] | X | X |
| *COPS3* [[3](#page6)] |  |  |
| *COPS4* [[3](#page6)] | X |  |
| *COPS8* [[3](#page6)] | X | X |
| *REL* (encodes *c-REL* TF) [[8](#page6)] |  |  |
| *CUX1* [[13](#page7)] | X | X |
| *DHX8* [[3](#page6)] |  | X |
| *SMAC* + *TRAIL2* [[14](#page7), [15](#page7)] | X |  |
| *EIF3C* [[3](#page6)] |  |  |
| *EIF3G* [[3](#page6)] | X |  |
|  |  |  |

***^1^The datasets that were incorporated in our pipeline***

*FBL* [[3](#page6)] *FIP1L1* [[3](#page6)]

*GATA2* [[4](#page6), [16](#page7), [17](#page7)]

*GSPT1* [[3](#page6)] *HNRNPC* [[3](#page6)]

*IL8* [[18](#page7)]

*JAK1* [[3](#page6)]

*KIF2C* [[3](#page6)]

*LDHA* (only under hypoxia conditions) [[19](#page7)]

*MAP3K7* [[20](#page7)]

*METAP1* [[3](#page6)]

*MIS18A* [[3](#page6)]

*NAE1* [[3](#page6)]

*NEDD8* [[3](#page6)]

*NFKB* pathway [[8](#page6)]

*NOL56* [[3](#page6)]

*NXF1* [[3](#page6)]

*OIP5* [[3](#page6)]

*PI3K-AKT-mTOR* pathway [[21](#page7), [22](#page7)]

*PKCδ* (*PRKCD*) [[23](#page7)] *PLK1* [[3](#page6)]

*PSMA5* [[3](#page6), [4](#page6)]

*PSMB5* [[3](#page6), [4](#page6)]

*PSMB6* [[3](#page6), [4](#page6)]

*PSMD14* [[4](#page6)] *RALB* [[8](#page6)] *SAE1* [[3](#page6)] *SIAH2* [[18](#page7)] *SMAD1* [[20](#page7)] *SMC4* [[3](#page6)] *SNAI2* [[24](#page7)] *STK33* [[25](#page7)]

*SYK* [[26](#page7)]

*TBK1* [[8](#page6)]

*THOC1* [[3](#page6)]

*TOP1* [[4](#page6)]

*TOP2A* [[4](#page6)]

*TPX2* [[3](#page6)] *TWIST1* [[27](#page8)] *UBA1* [[3](#page6)] *UBA2* [[3](#page6)] *UBE2I* [[3](#page6)] *USP39* [[3](#page6)] *VDAC3* [[28](#page8)]

*WT1* (Hugo symbol: *PAWR*) [[29](#page8)]

X X

X [***^2^***](#page2)

X X

X

X X

X

X

X X X X

X

X X X

X X

X X X X X

X

X

X X

X X

X X X

X X X X

X

X X X X

X X

***^2^Although GATA2 comes from a publication from which some data were used in this work, GATA2 was evaluated on an independent dataset too.***

Table [1](#page1) contains published *KRAS* SLPs in alphabetic order, along with the respective publication. Many of them consist of secondary screen findings as there is just a hint in the respective publication that they may be *KRAS* SLPs.
